# Extinction and emergence of genomic haplotypes during the evolution of *Avian coronavirus* in chicken embryos

**DOI:** 10.1590/1678-4685-GMB-2019-0064

**Published:** 2020-04-22

**Authors:** Paulo E. Brandão, Aline S. Hora, Sheila O. S. Silva, Sueli A. Taniwaki, Mikael Berg

**Affiliations:** 1 Universidade de São Paulo, Faculdade de Medicina Veterinária e Zootecnia, Departamento de Medicina Veterinária Preventiva e Saúde Animal, São Paulo, SP, Brazil.; 2 Universidade Federal de Uberlândia, Faculdade de Medicina Veterinária, Uberlândia, MG, Brazil.; 3 Swedish University of Agricultural Sciences, Department of Biomedical Sciences and Veterinary Public Health, Section of Virology, Uppsala, Sweden.

**Keywords:** Avian coronavirus, haplotype, mutant spectrum, NGS, evolution

## Abstract

*Avian coronavirus* (AvCoV) is ubiquitously present on poultry as a multitude of virus lineages. Studies on AvCoV phenotypic traits are dependent on the isolation of field strains in chicken embryonated eggs, but the mutant spectrum on each isolate is not considered. This manuscript reports the previously unknown HTS (high throughput sequencing)-based complete genome haplotyping of AvCoV isolates after passages of two field strains in chicken embryonated eggs. For the first and third passages of strain 23/2013, virus loads were 6.699 log copies/ μL and 6 log copies/ μL and, for 38/2013, 5.699 log copies/μL and 2.699 log copies/μL of reaction, respectively. The first passage of strain 23/2013 contained no variant haplotype, while, for the third passage, five putative variant haplotypes were found, with > 99.9% full genome identity with each other and with the dominant genome. Regarding strain 38/2013, five variant haplotypes were found for the first passage, with > 99.9% full genome identity with each other and with the dominant genome, and a single variant haplotype was found. Extinction and emergence of haplotypes with polymorphisms in genes involved in receptor binding and regulation of RNA synthesis were observed, suggesting that phenotypic traits of AvCoV isolates are a result of their mutant spectrum.

## Introduction

The evolution of RNA viruses must be assessed from the perspective of the quasispecies, defined as population of genomes linked through mutation with each other and with the genome of higher frequency, i.e., the dominant sequence, all of the variant and the dominant genomes resulting in a mutant spectrum that interacts at the functional level and works as a selection unit (Lauring and Andino, 2009). Structurally, each individual genome in the spectrum is defined by a collection of alleles in different sites occurring on the same individual or genome, which is known as the haplotype, including the variant and the dominant haplotypes.

The viral species *Avian coronavirus* or AvCoV (*Nidovirales*: *Coronavirida*e: *Coronavirinae*: *Gammacoronavirus*) is an enveloped virus of 120 nm in diameter. Its positive single-stranded RNA of circa 27 kb in length is subjected to an evolutionary rate (substitutions/site/year) of a 10^-3^ magnitude (Marandino *et al.*, 2015), which supports the radiation of AvCoV in six genotypes and 34 lineages (Valastro *et al.*, 2016; Jiang *et al.*, 2017).

AvCoV lineages show a variable pathogenicity and tropism and are the cause for the acute and chronic presentations of the highly contagious disease of poultry avian infectious bronchitis in its natural host *Gallus gallus* (Cook *et al.*, 2012). The control of this disease is based on a plethora of vaccine strains produced in embryonated chicken eggs.

The 5’ 2/3 of AvCoV genome code for the replicase complex is composed by non-structural proteins 2 to 16 arranged in ORF1ab, followed by genes for the spike envelope glycoprotein S (with the S1 and S2 domains), envelope E, membrane M and nucleocapsid N. Accessory proteins 3a,b genes are located upstream of E and 5a,b, and protein X genes are upstream of N genes, respectively. Untranslated regions (UTRs) are found at both the 5’ and 3’ ends of the genome (Cavanagh, 2007; Gomaa *et al.*, 2008).

The isolation of field strains of AvCoV in chicken embryonated eggs is a preliminary procedure for a series of downstream applications, such as diagnosis of avian infectious bronchitis, virus attenuation for vaccine manufacture, production of challenge virus for vaccine trials, pathogenicity and tropism assays, and complete genome sequencing. Nonetheless, the mutant spectra of isolates are not considered in most studies, and the phenotypic traits of AvCoV strains are explained based solely on the dominant sequences.

AvCoV quasispecies has been tentatively assessed using molecular cloning of partial or complete genes, followed by Sanger sequencing and comparison of polymorphic sites among sequences, or visual checking for multiple peaks in chromatograms without cloning (van Santen, 2008; Gallardo *et al.*, 2010; Saraiva *et al.*, 2018). However, these approaches are less sensitive for the detection of variant states of nucleotide sites when compared to HTS (High Throughput Sequencing) (Gregori *et al.*, 2014) and, more importantly, do not allow for the prediction of alleles that co-occur on a same segment or complete genome, as haplotyping can do.

Considering the absence to date of reports on full genome haplotypes for coronaviruses based on HTS and the need for a more complete characterization of AvCoV isolates in chicken embryos, this study was designed to (a) describe the mutant spectra for AvCoV strains based on full genomes haplotypes assembly, and (b) assess the mutant spectra after three passages in chicken embryos.

## Materials and Methods

### Origin and isolation of AvCoV strains

Strains GammaCoV/AvCoV/*Gallus gallus*/Brazil/23/2013 and GammaCoV/AvCoV/*Gallus gallus*/Brazil/38/2013 (from now on referred to simply as 23/2013 and 38/2013) were obtained from kidneys and cecal tonsils, respectively, collected in 2013 from chickens from two different farms in Brazil. AvCoV PCR screening and Sangersequencing spike typing done directly on pools of these organs were performed as in Cavanagh *et al.* (2002) and Worthington *et al.* (2008), respectively. The two strains were typed as GI-11 according to the classification in Valastro *et al.* (2016).

Suspensions of the respective field samples were submitted to isolation in embryonated SPF chicken eggs via inoculation on the allantoic route, and the second and third passages were prepared with the allantoic fluid from the previous passage. All passages were monitored for AvCoV, with the PCR described by Cavanagh *et al.* (2002).

The first (P1) and third (P3) passages were selected for the downstream analyses because three passages are recommended for AvCoV isolation (OIE, 2018), and the first passage would provide a truer picture of AvCoV sequence data as compared to the original chicken sample to assess the closest-to-wild AvCoV populations.

The study was approved by the Ethics Committee on the Use of Animals of the School of Veterinary Medicine, University of São Paulo under the register #2174/2011.

### Determination of virus load

The number of AvCoV genome copies in allantoic fluids harvested up to 72 h post-inoculation of P1 and P3 of strains 23/2013 and 2013/38 was obtained with a quantitative RT-PCR (qRT-PCR) based on SYBR green detection system, with primers reported by Callison *et al.* (2006) for the 5’UTR, with absolute genome quantifications/ μL cDNA obtained by comparison with a ten-fold dilution standard curve, ranging from 10^1^ to 10^7^ copies of a plasmid containing the target. All reactions were run in duplicate.

### Full genomes High Throughput Sequencing (HTS)

Allantoic fluids (strains 23/2013 and 38/2013, P1 and P3) were clarified by centrifugation at 16,000 x *g* for 3 min at4 ºC, filtered through 0.45 μm syringe filters, and treated with DNase-free RNase. Total RNA was then extracted with Trizol Reagent (Life Technologies) and RNeasy Mini kit (Qiagen), and used with Superscript III and Klenow exo-DNA polymerase (Life Technologies) to obtain random ds-cDNAs.

Libraries and sequencing kits were Nextera XT Index and Nextera XT DNA (Illumina), and reads were obtained with a NextSeq500 (Illumina) sequencer using the NextSeq500 Mid output v2 kit (2 x 150 bp). Read quality was assessed with FASTQC, and full genomes were assembled with CLC Genomics Workbench v. 11.0.1 (Qiagen), with the reference-mapping approach.

### Haplotype assembly

Paired and trimmed reads were clustered by Bayesian inference to assemble the putative global haplotypes for 23/2013 and 38/2013 P1 and P3 using PredictHaplo 1.0 (Prabhakaran *et al.*, 2014), with the respective dominant genome as reference and the following parameters: max reads in window 10,000; entropy threshold 4e^-2^; min mapping qual 30; min read length 64; max gap fraction 0:05 (relative to alignment length); min align score fraction 0:35 (relative to read length); alpha MN local 25; min overlap factor 0.85; local window size factor 0.7; max number of clusters 25; MCMC iterations 501; and deletions inclusion.

The number of polymorphic sites between passages and among the different coding regions was compared with the Fisher’s Exact Test at a significance level of 0.05 using the Easy Fisher Exact Test Calculator.

### Genealogical analysis

A Maximum Likelihood nucleotide tree with the TN model was built with MEGA X software (Kumar *et al.*, 2018), using 100 bootstrap replicates and 5 gamma categories for the full genomes of variant haplotypes and dominant sequences.

### Recombination analysis of haplotypes

Within-passages recombination analysis was run for both P1 and P3 of the two AvCoV strains dominant and variant haplotypes full genomes using RDP4 (Martin *et al.*, 2015) with the methods RDP, Geneconv, Chimaera, MaxChi, BootScan, SiScan, 3Seq, and LARD, with minimum *p*-value = 0.05.

## Results

### Virus loads

For the first and third passages of 23/2013, the virus loads (based on the number of genome copies) were 6.699 log copies/ μL and 6 log copies/ μL and, for 38/2013, 5.699 log copies/ μL and 2.699 log copies/ μL, respectively.

### Dominant complete genomes

Coverages for 23/2013 P1, 23/2013 P3, 38/2013 P1 and 38/2013 P3 were 4.613log, 4.829log, 4,784log and 5.431log, respectively.

The dominant complete genomes of 23/2013 P1 and P3 were 27,615 nt long and annotated as 5’UTR (nt 1 528), ORF1ab (529 20,357), spike S (20,308 23,817), 3a (23,817 23,990), 3b (23,990 24,181), envelope E (24,165 24,488), membrane M (24,457 25,137), X (25,138 - 25,422), 5a (25,497 25,694), 5b (25,691 25,939), nucleo-capsid N (25,882 27,111) and 3’UTR-poly-A tail (27,112 27,615). P1 and P3 nt identity was > 99.9% with a single, non-synonymous substitution on the spike (nt T732A/ aa N244K).

For 38/2013, P1 and P3 dominant complete genomes were 27,618 long annotated as 5’UTR (1 528), ORF1ab (529 20,363), S (20,314 23,823), 3a (23,823 23,996), 3b (23,996 24,187), E (24,171 24,494), M (24,463 - 25,143), X (25,144 25,428), 5a (25,503 25,700), 5b (25,697 25,945), N (25,888 27,114) and 3’UTR-poly-A tail (27,115 27,618). P1 and P3 nt identity was > 99.9% with a single nt substitution on the 3’UTR (nt G27,605T). 

P3 complete genomes of 23/2013 and 38/2013 can be found under the GenBank Accession numbers KX258195.1 and MG913342; 23/2013 P3 genome had been published already (Brandão *et al.*, 2016); first passages and variant haplotypes sequences were not submitted to the GenBank to avoid redundancy.

### Haplotypes

P1 of strain 23/2013 contained no variant haplotype, as only a dominant sequence was found, while for P3 five putative variant haplotypes were found, with > 99.9% full genome identity with each other and with the dominant genome.

Regarding strain 38/2013, five variant haplotypes were found in P1, with > 99.9% full genome identity with each other and with the dominant genome. A single variant haplotype, different from all P1 haplotypes, was found in P3, with > 99.9% identity with the dominant sequence.

Table 1 shows the signatures of each haplotype and the respective (if any) amino acid substitution regarding the dominant sequence as a reference. P1 of 38/2013 showed the highest (n = 70) number of polymorphic sites, with significant higher numbers (*p-*values ranging from 0.0048 to < 0.00001) in X, IG (intergenic region between M and 5a), 5a and 5b, and with further polymorphic sites in the 5’UTR, nsps3, 14-16, S, 3a, and N. Premature stop codons were in 3a in five and 5a in four variant haplotypes, while only three polymorphic sites, all in the 3’UTR, were found for P3, with a significant (*p* < 0.00001) drop in the number of variable sites between these two passages. For 23/2013 P3, eight polymorphic positions were found among the five variant haplotypes (nsps 5-6 and 9, S, E and 3’UTR), with a significant higher number in nsp9 only (*p* < 0.00001), with no premature stop codons. 

**Table 1:** Haplotypes (H) found for passages 1 (P1) and 3 (P3) of AvCoV strain 38/2013 and 3 (P3) of strain 23/2013 for each position based on the dominant sequence for each passage and the respective (if any) amino acid substitution; syn=synonymous mutations; *= stop codon; NC=non-coding region; del=deletion; #=haplotype number not found; aa= aa synonymous (syn) or non-synonymous mutations and the positions regarding the specific protein; Dots indicate identity with the dominant sequence. Same haplotype numbers do not indicate identical haplotypes for different strains and passages.

	Position in dominant	Dominant	H1	H2	H3	H4	H5	aa
**38/2013 P1** 5’UTR	170	C	.	T	.	G	.	NC
nsp3	3867	A	.	.	.	.	T	syn440
	3895	A	.	.	.	.	C	M450L
	3917	A	.	.	.	.	G	K457R
	3936	T	.	.	.	.	C	syn463
	3957	T	.	.	.	.	C	syn470
	5707	C	.	.	T	.	.	R1054C
nsp14	17825	T	G	G	G	G	G	N316K
nsp15	18943	T	A	A	A	A	A	V168D
nsp16	19555	T	A	A	A	A	A	L34Y
	19556	A	C	C	C	C	C	L34Y
	20195	A	T	T	T	T	T	syn247
S	20500	T	C	C	C	C	C	S63P
	20598	T	C	C	C	C	C	syn95
	20601	A	G	G	G	G	G	syn96
	21054	T	A	A	A	A	A	D247E
	21198	A	G	G	G	G	G	syn295
	21864	C	G	G	G	G	G	syn517
	22374	A	C	C	C	C	C	syn687
	22380	G	A	A	A	A	A	syn689
	22476	G	A	A	A	A	A	syn721
	22587	C	T	T	T	T	T	syn758
	22590	T	C	C	C	C	C	syn759
	22750	C	G	G	G	G	G	H813D
	23028	C	G	G	G	G	G	syn905
3a	23837	A	C	del	del	del	del	T6H; *
X	25188	T	C	C	C	C	C	synA15
	25293	C	A	A	A	A	A	F50L
	25373	G	A	A	A	A	A	R77N
	25374	A	C	C	C	C	C	R77N
	25377	A	C	C	C	C	C	syn78
	25401	T	G	G	G	G	G	S86R
	25402	C	T	T	T	T	T	syn87
	25411	A	C	C	C	C	C	S90H
	25412	G	A	A	A	A	A	S90H
	25422	A	T	T	T	T	T	K93N
	25423	A	G	G	G	G	G	N94D
	25428	G	A	A	A	A	A	syn*
IG	25430	A	T	T	T	T	T	NC
	25431	T	G	G	G	G	G	NC
	25432	A	G	G	G	G	G	NC
	25444	C	T	T	T	T	T	NC
	25448	A	C	C	C	C	C	NC
	25449	C	T	T	T	T	T	NC
	25458	T	G	G	G	G	G	NC
	25461	C	A	A	A	A	A	NC
	25462	A	T	T	T	T	T	NC
	25465	A	G	G	G	G	G	NC
	25473	T	G	G	G	G	G	NC
	25474	A	G	G	G	G	G	NC
	25476	G	C	C	C	C	C	NC
	25484	A	C	C	C	C	C	NC
	25485	A	G	G	G	G	G	NC
	25494	G	A	A	A	A	A	NC
	25498	G	A	A	A	A	A	NC
5a	25504	T	G	G	G	G	G	*
	25672	T	A	A	A	A	A	*
	25674	T	A	A	A	A	A	*
5b	25714	A	T	T	T	T	T	E6D
	25726	T	C	C	C	C	C	syn10
	25748	C	A	A	A	A	A	syn18
	25783	T	C	C	C	C	C	syn29
	25831	T	C	C	C	C	C	syn45
	25843	G	A	A	A	A	A	syn49
	25860	A	T	T	T	T	T	E55V
	25861	G	A	A	A	A	A	E55V
N	26122	C	G	G	G	G	G	P79A
	26412	T	C	C	C	C	C	syn175
	26911	A	T	T	T	T	T	T342S
3’UTR	27333	T	G	G	G	G	G	NC
**38/2013 P3**								
3’UTR	27605	T	G	#	#	#	#	NC
	27607	T	_	#	#	#	#	NC
	27608	A	_	#	#	#	#	NC
**23/2013 P3**								
nsp5	9260-9261	GT	.	.	.	CA	CA	G153A
	9281	T	.	.	.	A	A	M160K
nsp6	10516	C	A	A	.	.	.	P265T
nsp9	11599	G	C	C	.	.	.	V39F
	11657	A	.	.	.	G	G	E58G
S	21039	A	.	T	T	.	T	K244N
E	24459	G	.	T	T	.	T	V99F
3’UTR	27602	T	.	G	G	.	G	NC

**Figure 1:**
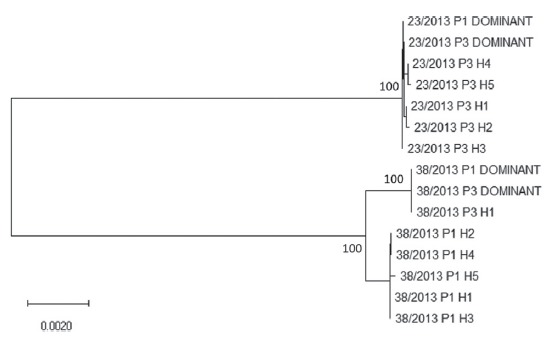
Full genome Maximum Likelihood tree based on the TamuraNei model for variant haplotypes (H) and dominant sequences of AvCoV strains 23/2103 and 38/2013 P1 and P3. The tree with the highest log likelihood (-41,512.06) is shown. A discrete Gamma distribution was used to model evolutionary rate differences among sites [5 categories (+G, parameter = 0.7532)]. The rate variation model allowed for some sites to be evolutionarily invariable ([+I], 77.34% sites). The tree is drawn to scale, with branch lengths measured in the number of substitutions per site. The numbers at each node are bootstrap values (only values = 100 are shown); the bar represents the number of substitutions per nucleotide site.

A genealogy of variant haplotypes and dominant sequences for 23/2013 and 38/2013 P1 and P3 is displayed in Figure 1, which shows the segregation in two main strainspecific clusters.

No recombination was found for 23/2013 P3 an 38/2013 P1 haplotypes. Recombination analysis for 23/2013 P1 and 38/2013 P3 was not possible, as the number of sequences to be analyzed was only one and two, respectively.

## Discussion

For both AvCoV strains used in this study, virus loads decreased from the first to the third passage, but a more marked drop was evident for 38/2013, as a 3-log decrease was found from P1 to P3, while, for 23/2013, the decrease was of 0.699 log.

The genomic nucleotide identities between P1 and P3 dominant sequences was high (> 99.9%) for both strains, with a unique substitution in both cases. While for 38/2013 the substitution occurred in the 3’UTR, for 23/2013 a nonsynonymous substitution led to a change ofaN toa K, both hydrophilic amino acids, in residue 244 of the spike gene, within the 253 N-terminal amino acids of the spike glyco-protein. This had already shown to be required for receptor binding (Promkuntod *et al.*, 2014).

A more complex evolutionary pattern was found, however, after haplotype analysis, as, for instance, all of the 13 S gene polymorphisms present in the five 38/2013 P1 variant haplotypes (Table 1) have been extinguished on P3 and a single mutation (A732T/K244N, position 21039 regarding the genome) emerged on variant haplotypes H2-3 and 5 of 23/2013 P3, preserving the state found in P1 dominant sequence.

Mutations on the S1 subunit of spike glycoprotein gene have already been described as being related to adaptation of AvCoV during passages from chickens to embryos and back (Cavanagh *et al.*, 2005; Geerligs *et al.*, 2011; Leyson *et al.*, 2016). The α2,3-linked sialic acid has been shown to be the cell receptor for the spike glycoprotein of AvCoV (Winter *et al.*, 2006) and is present on the chorioallantoic membranes (CAM) of chicken embryos, while the α2,6-linked arrangement is not (Ito *et al.*, 1997).

The presence of α2,6-linked sialic, lacking on CAM but co-existing with the α2,3 arrangement in adult chickens, has been suggested to hava a role in the selection of amino acid substitutions during serial passages of AvCoV from embryos to chickens (Leyson *et al.*, 2016). Thus, the low diversity on spike genes at passage 3 might have been a consequence of both purifying (regarding strain 38/2013) and positive selection (in the case of the single variant haplotype and the N244K found on 23/2013 P3), leading to fine tuning of the affinity of the mutant spectra to CAM α2,3-linked sialic acid. As the receptor type is the main difference between embryos and adult chickens, receptordriven variant haplotypes selection might be a major force for AvCoV mutant spectrum evolution during isolation in egg, as attachment is the primary step for the intracellular virus life cycle.

Though the 5’ and 3’UTRs have a role on RNA transcription and replication, all SNPs on the variant haplotypes and dominant sequences occurred outside the conserved octamer described for AvCoV (nt positions 27,527-27,534) and of the stem-loop like motif (sm2, nt 27,471-27,511) involved in RNA replication, both on the 3’UTR, and out of the leader sequence region involved in subgenomic RNA synthesis on the5’UTR (Hsue and Masters, 1997; Masters, 2006). This makes any gain or lossof-function unlikely in this case, favoring genetic drift as an evolutionary force, similarly to the observed for the also non-coding intergenic (IG) region between M and 5a that also accumulated several SNPs in P1 only of 38/2013 (n = 17) in all five variant haplotypes.

Variant haplotypes H1-H5 of 38/2013 P1 accumulated SNPs on nsps in ORF1ab that were extinguished in P3, while SNPs in these areas emerged in four of the five variant haplotypes in 23/2013 P3 (Table 1). Considering the role of these nsps in RNA replication and transcription, and that mutations on the replicase genes have already been shown to occur after serial passages in embryos and are related to attenuation of virulent strains of AvCoV (Ziebuhr and Snijder, 2007; Albanese *et al.*, 2018), these mutations could also have resulted in an initial degree of attenuation of these two strains at passage 3.

Proteins 3a and 5a, for which premature stop codons were found for variant haplotypes H2-5 and 1-5, respectively, of 38/2013 P1, were reported as nonessential for AvCoV replication, though a lower virulence has been reported after the deletion of accessory genes 3 and 5 (Laconi *et al.*, 2018). Thus, a truncated 3a and 5a and SNPs in 5b, also found in all variant haplotypes of 38/2013 P1, would not abolish the replication of these haplotypes but could account for the decrease in virus loads.

ORF X, also called 4b, has been shown to code for an accessory protein of 11kDa (Bentley *et al.*, 2013), but, as all polymorphisms on this ORF have experienced extinction between P1 and P3 of 38/2013, it is under a selection regime similar to that of a structural protein, like the spike glycoprotein. Hence, an essential role for X on the AvCoV life cycle should be further investigated.

A unique SNP on the E protein gene leading to a V99F amino acid substitution emerged on three variant haplotypes at passage 3 of 23/2013 (Table 1). E is a core protein, as well as M, for the assembly of the virion (Masters *et al.*, 2006), and is thus expected to present low polymorphism due to purifying selection.

The nucleocapsid protein N is subjected to a more intense selection pressure as a result of stronger structural constraints, due to its intricated conformational and charge-based interactions with the genomic RNA on the nucleocapsid and interaction with nsp3 on the cytoplasm during RNA replication (Masters, 2006; Hurst *et al.*, 2013). This evolutionary pattern could explain the low (two nonsynonymous and one synonymous) number of polymorphisms found for this gene, detected exclusively on haplotypes H1-H5 of 38/2013 P1.

The evolution of 23/2013 and 38/2013 AvCoV strains after three passages in chicken embryos did not result in shared nucleotides states that could be considered as attenuation markers. This is also illustrated by the absence of convergent evolution as noticed in Figure 1, as no haplotypes crossed the strain-specific clades limit at passage 3.

Significant AvCoV quasispecies differences occur in a same chicken during infection regarding the diverse organs in which the virus replicates (van Santen and Toro, 2008; Gallardo *et al.*, 2010). Hence, differences in starting mutant spectra populations resulting from different origins for the two strains used in this study, i.e, kidneys (23/2013) and cecal tonsils (38/2013), as well as in virus loads present on the starting inocula, before P1 (not measured in this experiment), could account for the different evolutionary paths, resulting in non-coincident polymorphic sites.

Applying Sanger sequencing to complete or partial genes to assess diversity based on molecular cloning or chromatograms and even NGSs complete genomes variant analyses are deeply reductionist approaches compared to complete genome haplotype analyses, which enable a more accurate characterization the mutant spectra. Understanding the mutant spectra must be considered not only for studies on AvCoV evolution, but also those focused on the virus pathogenesis, vaccinology and diagnosis.

As a conclusion, the evolution of AvCoV haplotypes in chicken embryos after a low passage number, as used in isolation routine, results in a reduced fitness with a lowered diversity essentially in genes that regulate virus RNA synthesis and attachment to cell receptors.
